# Vaccine-draining lymph nodes of cancer patients for generating anti-cancer antibodies

**DOI:** 10.1186/s12967-017-1283-8

**Published:** 2017-08-29

**Authors:** Girja S. Shukla, Walter C. Olson, Stephanie C. Pero, Yu-jing Sun, Chelsea L. Carman, Craig L. Slingluff, David N. Krag

**Affiliations:** 1 Department of Surgery & Vermont Cancer Center, Larner College of Medicine, University of Vermont, Given Bldg Rm E309, Burlington, VT 05405 USA; 2 Human Immune Therapy Center, University of Virginia, Charlottesville, VA 22908 USA

**Keywords:** Cancer vaccine, B cells, Antibodies, Melanoma, Human

## Abstract

**Background:**

Our research is focused on using the vaccine draining lymph node to better understand the immune response to cancer vaccines and as a possible source of anti-cancer reagents. We evaluated vaccine draining lymph nodes archived from a clinical study in melanoma patients and determined the reaction of B cells to the vaccine peptides.

**Methods:**

Mononuclear cells (MNCs) were recovered from cryopreserved lymph nodes that were directly receiving drainage from multi-peptide melanoma vaccine. The patients were enrolled on a vaccine study (NCT00089219, FDA, BB-IND No. 10825). B cell responses in the vaccine-draining lymph nodes were studied under both stimulated and un-stimulated conditions. Cryopreserved cells were stimulated with CD40L, stained with multiple human cell-surface markers (CD19, CD27, IgM) to identify different categories of B cell sub populations with flow cytometry. Hybridomas were generated from the lymph node cells after CD40L-stimulation. Cells were fused to murine plasmacytoma P3X63.Ag8.653 using Helix electrofusion chamber. ELISA was used to evaluate hybridoma derived antibody binding to vaccine peptides.

**Results:**

Viable MNCs were satisfactorily recovered from lymph nodes cryopreserved from six vaccine study patients 8–14 years previously. B cell ELISPOT demonstrated responses for each patient to multiple vaccine peptides. CD40L stimulation of lymph node cells increased the proportion of CD19^+^ CD27^+^ cells from 12 to 65% of the sample and increased the proportion of class-switched cells. Screening of IgG secreting clones demonstrated binding to melanoma vaccine peptides.

**Conclusions:**

B cells were successfully recovered and expanded from human cryopreserved vaccine-draining lymph nodes. Individual B cells were identified that secreted antibodies that bound to cancer vaccine peptides. The ability to reliably generate in vitro the same antibodies observed in the blood of vaccinated patients will facilitate research to understand mechanisms of human antibody activity and possibly lead to therapeutic antibodies.

## Background

Our research is focused on using lymph nodes receiving drainage from a cancer or a vaccine to better understand the immune response and to generate new anti-cancer reagents. Debris from a tumor or interstitial injection of antigens are absorbed into lymphatic ducts and transported to regional lymph nodes. It is in the lymph node that antigen is processed and the initial immune response occurs. The development of lymphatic mapping with radioactive tracers allows precise localization of the draining lymph nodes from almost any tissue in the body [1]. In a series of clinical vaccine studies at the University of Virginia (UVA), a draining lymph node was used for evaluating the T cell response [2, 3]. Radiotracer lymphatic mapping was used to identify the lymph node receiving drainage from the vaccine and the lymph node was removed with minimal morbidity [4]. Analysis of the T cell response in the vaccine-draining lymph nodes was more sensitive than analysis of the circulating T cell response in the peripheral blood [4, 5]. Findings related to T cells in the node correlated with systemic findings. For example, CD4^+^ T cell responses were positively associated with serum antibody titers to the vaccine peptides [2]. It was prescient for the investigators of the UVA vaccine study to cryopreserve residual lymph node mononuclear cells (MNCs) and other clinical material for future research.

In the UVA study as well as other vaccine studies, positive clinical outcomes were associated with elevated serum titers of anti-vaccine antibodies [2, 6, 7]. In animals, vaccine-induced antibodies can prevent tumor growth and even eradicate existing bulky tumors [8]. Characterization of these antibodies shows that they exhibit an extensive range of functional activity. Further study of vaccine-induced antibodies will be valuable to better understand how antibodies interact with other immune effector mechanisms to inhibit the tumor. Unfortunately, there is not yet a reliable method to produce antibodies generated by human cancer patients that have received a cancer vaccine. The data presented here is focused on antibody secreting cells present in vaccine-draining lymph nodes. We hypothesized that B cells producing antibodies to the vaccine peptides may be detected ex vivo from the cryopreserved lymph node MNCs. These cryopreserved MNCs archived from the UVA vaccine trial were used for the study presented here to identify individual B cells that secreted anti-vaccine antibodies.

## Methods

### Subjects and vaccination

The patients were enrolled on a vaccine study (FDA, BB-IND No. 10825, and UVA institutional review board HIC No. 10464, NCT00089219) and had received a series of vaccinations with 6 different melanoma related peptides. Patients with stage IIIB to IV melanoma and expressed at least one of the five HLADR alleles by which CD4 T-cell recognition of the vaccine peptides had been defined were included in the study. Patients received vaccine comprised of 6 melanoma peptides namely, AQN (AQNILLSNAPLGPQFP), FLL (FLLHHAFVDSIFEQWLQRHRP), RNG (RNGYRALMDKSLHVGTQCALTRR), TSY (TSYVKVLHHMVKISG), LLK (LLKYRAREPVTKAE), WNR (WNRQLYPEWTEAQRLD) and tetanus helper peptide (AQYIKANSKFIGITEL). Melanoma peptides were derived from Tyrosinase (AQ and FLL), Melan-MART1 (RNG), MAGE-1, MAGE-2, MAGE-3, MAGE-6 (TSY and LLK), and gp100 (WNR) proteins. The mixture of these six class II major histocompatibility complex-restricted peptides were administered in 1 mL Montanide™ ISA-51 (Seppic, Fairfield, NJ) adjuvant emulsion of 110 µg granulocyte macrophage colony-stimulating factor (Schering-Plough, Kenilworth, NJ). The vaccinations at days 1, 8, 15 were divided between two injection sites (arm and thigh). At each injection site, half of the vaccination was administered subcutaneously, and half was administered intradermally. The details of the patient selection, vaccine preparation and immunization have been previously described [4]. Samples were selected for the current study that had sufficient archived material to conduct the analyses.

### Lymph node localization and removal

The lymph node draining the thigh vaccine was localized by lymphatic mapping with Tc^99^ sulfur colloid approximately 1 week after the third vaccination [9]. Selective biopsy of the lymph node was performed under local anesthesia with the intra-operative aid of a sterile hand-held gamma probe (Care Wise, Morgan Hill, CA). The incision was routinely 1 in. long, and the node was removed in the out-patient clinic. There were no infections or complications at these surgical sites.

### Lymph node processing

The surgically excised lymph node was placed in a culture disk containing RPMI medium with 10% fetal calf serum and minced into small pieces (2–3 mm) with sterile scalpel and scissors. The cells along with tissue fragments were filtered through 60 μm filter. The unfiltered tissues in the filter was gently grinded with the help of rubber end of 1-mL syringe plunger with intermittent rinsing with the medium. The grinding of fragments across the mesh facilitated tissue breaking and cell liberation. The cells collected by centrifugation at a speed of 300*g* for 5 min were treated with lysis buffer for 5 min to remove erythrocytes. Following two washes with the medium, the cells were cryopreserved in 90% fetal calf serum and 10% DMSO. Archived samples from 6 patients were selected for the present study.

### B-cell ELISPOT

B-cell responses in the vaccine-draining lymph nodes were studied under both stimulated and un-stimulated conditions as described previously [10]. Briefly, the MNCs from the lymph node were stimulated for 3 days with R-848 (Mabtech, Inc., Cincinnati, OH) and IL-2 (PeproTech, Rocky Hill, NJ), before transferring them to Multiscreen IP-PVDF filter microplates (Millipore) coated with affinipure goat anti-Human IgG Fcγ Fragment Specific (Jackson Immuno Research, West Grove PA) or vaccine peptides (2 μg total, individual or a mixture of all the six), diluted in PBS, for determining the total number of IgG-secreting B cells and anti-vaccine peptide antibody-secreting B cells, respectively. The irrelevant synthetic peptide used had the following sequence NH_2_-RVQECKYLYYDNDYLCKDDG-OH. Plates were washed 3 times with R10 media (RPMI + 10% fetal bovine serum) and then blocked for 2–4 h at 37 °C in R10 media. Following stimulation, MNCs were allowed to settle to the bottom of the coated filter plate wells as a monolayer and incubated overnight. The cells were rinsed off the filter plate and antibody spots representing the secretion of antibody were detected with biotin-SP-affiniPure goat anti-human IgG, Fcγ (Jackson Immuno Research) and streptavidin–alkaline phosphatase conjugate (Sigma, St. Louis, MO) and visualized with SIGMAFAST 5-Bromo-4-chloro-3-indolyl phosphate/Nitro blue tetrazolium (Sigma) for 3–5 min until spots developed. Antibodies were diluted in 50 mM tris-buffered saline (pH 7.4) containing 0.05% tween-20 (TBST-0.05%) and 1% FBS. All washes were done with TBST-0.05% with the exception of the TBS only wash just prior to substrate addition. Fully dried membranes were imaged using the DigiDoc-It Imaging System (UVP, LLC, Upland, CA).

### CD40L-stimulated amplification

The cryopreserved MNCs from vaccine-draining lymph nodes were used for separating CD27^+^ cells using CD27 magnetic beads (Miltenyi Biotec, Auburn, CA). CD27^+^ cells were cultured [11] for 5 days over a monolayer of irradiated tCD40L cells (NIH-3T3-hCD40 ligand cells, provided by Dr. Gordon J. Freeman, Dana-Faber Cancer Institute, Boston, MA) in presence of IL-4, and IL-10 (Thermo Scientific, Rockford, IL) in IMDM supplemented with 10% human serum and cyclosporine A (Sigma-Aldrich, St Louis, MO) [12]. Two rounds of amplifications were done and following each round the cells were counted and analyzed by flow cytometry.

### Flow analysis of cells from the vaccine-draining lymph nodes

The CD40L-stimulated lymph node cells in PBS containing 2% fetal bovine serum were stained with fluorophore-conjugated antibodies (BD Biosciences, San Jose, CA) against specific human cell-surface markers (CD19, CD27, IgM) to identify different categories of B cell sub populations. The stained cell samples were run on BD LSRII (BD Biosciences, San Jose, CA) flow cytometer equipped with BD FACSDiva™ version 8 software for data acquisition. The data were analyzed using FlowJo™ v10.1 software (FlowJo, LLC, Ashland, OR) following required gating with the help of proper controls including fluorescence minus one. This helped to determine the effect of CD40L-stimulated amplification on the recovery of mature class-switched (IgM^−^ CD^19+^ CD27^+^) memory B cells.

### Hybridoma generation

Hybridomas were generated from the lymph node cells that had been subjected to 2 rounds of amplification with CD40L-stimulation. The hybridoma fusion partner to the lymph node cells was murine plasmacytoma P3X63.Ag8.653 [13] from ATCC (Manassas, VA). CD40L-stimulated cells were fused under hypo-osmolar condition [14] using a Helix chamber (Eppendorf, Westbury, NY). Around 2500 fused cells were distributed per well in 96-well plates. Following HAT (hypoxanthine–aminopterin–thymidine medium, ATCC) selection, hybridomas were shifted to 1× HT (hypoxanthine–thymidine, ATCC) and eventually to a complete growth medium consisted of RPMI-1640 (Mediatech, Inc., Manassas, VA) containing 10% FBS, 0.1 mM non-essential amino acids (Lonza Walkersville, Inc., Walkersville, MD), 100 μg/mL streptomycin, 100 U/mL penicillin (Mediatech, Inc.) and 55 μM 2-mercaptoethanol (Thermofisher Scientific, Waltham, MA). This resulted in 0–1 growing hybridoma per well. The hybridomas were cultured in complete growth medium for several days for a detection of secreted antibody in the medium.

### Clone screening for antibody secretion and vaccine peptide binding

The hybridoma supernatants were screened by ELISA for identifying the immunoglobulin-secreting wells. Each immunoglobulin secreting clone was evaluated for binding to the vaccine peptides. Briefly, maxiSorp^®^ immuno™ flat-bottom 96 well plates (Thermo Fisher Scientific) were coated with goat anti-human IgG (H + L) antibody (Southern Biotech, Birmingham, AL) or 0.5 μg peptides in carbonate buffer (pH 9.6) and blocked with 1% casein solution in TBS (Thermo Fisher Scientific) for screening IgG-secreting wells and vaccine peptide binding antibodies by ELISA. The target-binding antibodies were detected with HRP-conjugated goat anti-human IgG (H + L) antibody (Thermo Fisher Scientific) and 3,3′,5,5′-tetramethylbenzidine soluble substrate (EMD Millipore, Darmstadt, Germany). The formation of blue-colored horseradish peroxidase product was read immediately at 650 nm for 15 min at 3-min intervals using a Synergy HT plate reader (BioTek Instruments, Inc., Winooski, VT).

## Results

The mononuclear cells from vaccine-draining lymph nodes of 6 patients (case numbers: VMM871, VMM701, VMM819, VMM683, VMM582, and VMM216) that were vaccinated with tumor antigen peptides were used for the analyses of this study. The counting of cryopreserved MNCs from these patients showed their high viability (85–96%) and presence of the required numbers for this study.

### B-cell ELISPOT analysis of cells from the vaccine-draining lymph nodes shows broad reactivity to multiple vaccine peptides

**Fig. 1:**
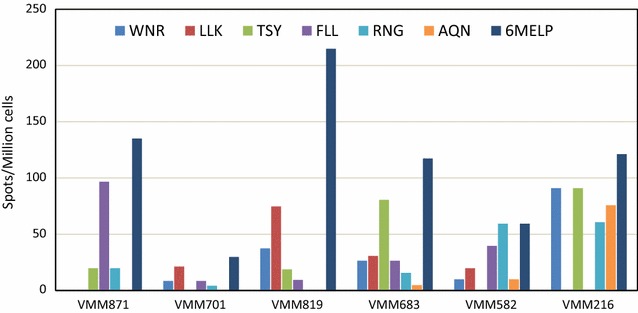
B cell ELISPOT analyses showing the number of vaccine-draining lymph node B cells per patient that secreted IgG Abs against individual and pooled (6MEL) vaccine peptides. The number of spots were plotted after subtracting the values of irrelevant peptide negative control

B cell ELISPOT analyses of MNCs recovered from vaccine-draining were conducted for each patient and the results calculated as antibody secreting B cells (spots)/million MNCs for each patient are presented in Fig. 1. B cell responses were studied for all the six vaccine peptides individually and for their mixture. All the six patients exhibited B cell responses against the mixture of six vaccine peptides (6MELP). The analyses of MNCs showed varying degree of B cell responses against WNR, FLL and RNG peptides in five patients, and against LLK, TSY and AQN peptides in four patients. The MNCs of two patients (VMM683, VMM582) exhibited B cell responses against all the six peptides, while the other four patients (VMM871, VMM701, VMM819, VMM216) had responses against only four vaccine peptides. The results showed broad B cell responses by the lymph node samples from patients against the vaccine peptides. However, it is interesting to note that the lymph node MNCs of patients reacted very differently to individual vaccine peptides. The patients VMM871, VMM701, VMM819, VMM683, VMM582 and VMM216 showed highest B cell responses against peptides FLL, LLK, LLK, TSY, RNG, and WNR-TSY peptides, respectively.

### CD40L-stimulation of cells from the vaccine-draining lymph nodes increases the proportion of class-switched B cell sub-population

**Fig. 2:**
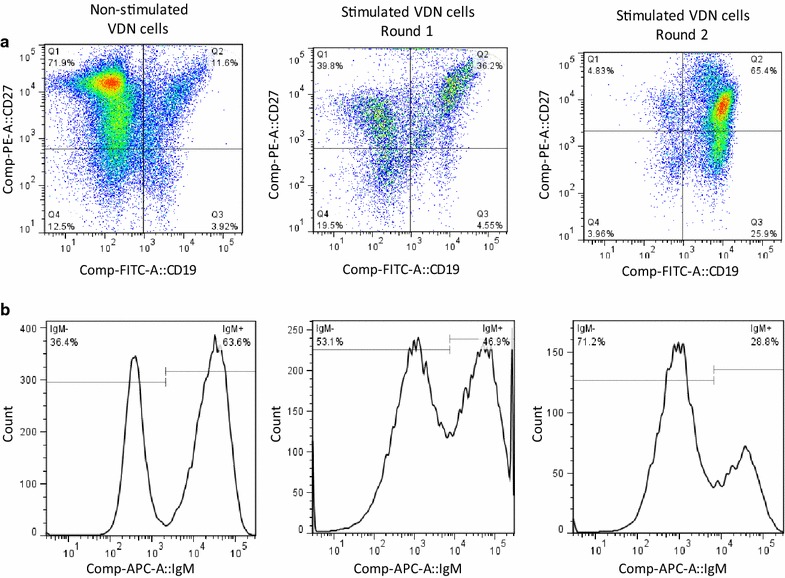
Representative density plots and histograms from flow cytometric analyses of vaccine-draining lymph node cells, showing enrichment of **a** CD19^+^CD27^+^ cells and **b** class-switched IgM^−^ CD19^+^ CD27^+^ cells following subsequent rounds of CD40L-stimulated amplification

The 2 rounds of CD40L-stimulated amplification of CD27^+^ cells from vaccine-draining lymph node resulted in ~10-fold amplification (round 1, ~fivefold; round 2, ~twofold) of total cells. The CD27^+^ cells with and without CD40L stimulation were stained with the selected cell surface markers and analyzed by multicolor flow cytometry. Lymphocytes were gated based on forward (FSC) and side (SSC) scatter characteristics, followed by CD19^+^ and CD27^+^ gating for B cells. The CD19^+^ and CD27^+^ lymph node cells were further gated for the analysis of IgM^+^ and IgM^−^ cells. The CD19^+^ CD27^+^ IgM^−^ gated cells represent the class-switched population of the B cells. The representative density plots from a sample of patient VMM683 in Fig. 2a show the subpopulations of CD19^+^ and CD27^+^ cells in unstimulated and CD40L-stimulated cells. The first round of CD40L stimulation of cells increased (12 vs 36%) the subpopulation of CD19^+^ CD27^+^ cells in the sample. The second round of stimulation further increased the proportions of CD19^+^ CD27^+^ cells to 65% in the sample. Further analysis of CD19^+^ CD27^+^ cells showed that CD40L-stimulation decreased the IgM^+^ cell subpopulation in lymph node cells. The representative histograms in Fig. 2b depict that the first round of CD40L-stimulation increased the class-switched CD19^+^ CD27^+^ IgM^−^ cell subpopulation from 36% in non-stimulated cell sample to 53% in CD40L-stimulated cells. The histogram following the second round of CD40L stimulation shows a further increase to 71% in the proportion of CD19^+^ CD27^+^ IgM^−^ cells, representing class-switched B cells.

### Hybridoma screening for IgG secreting clones and their binding to vaccine peptides

**Fig. 3:**
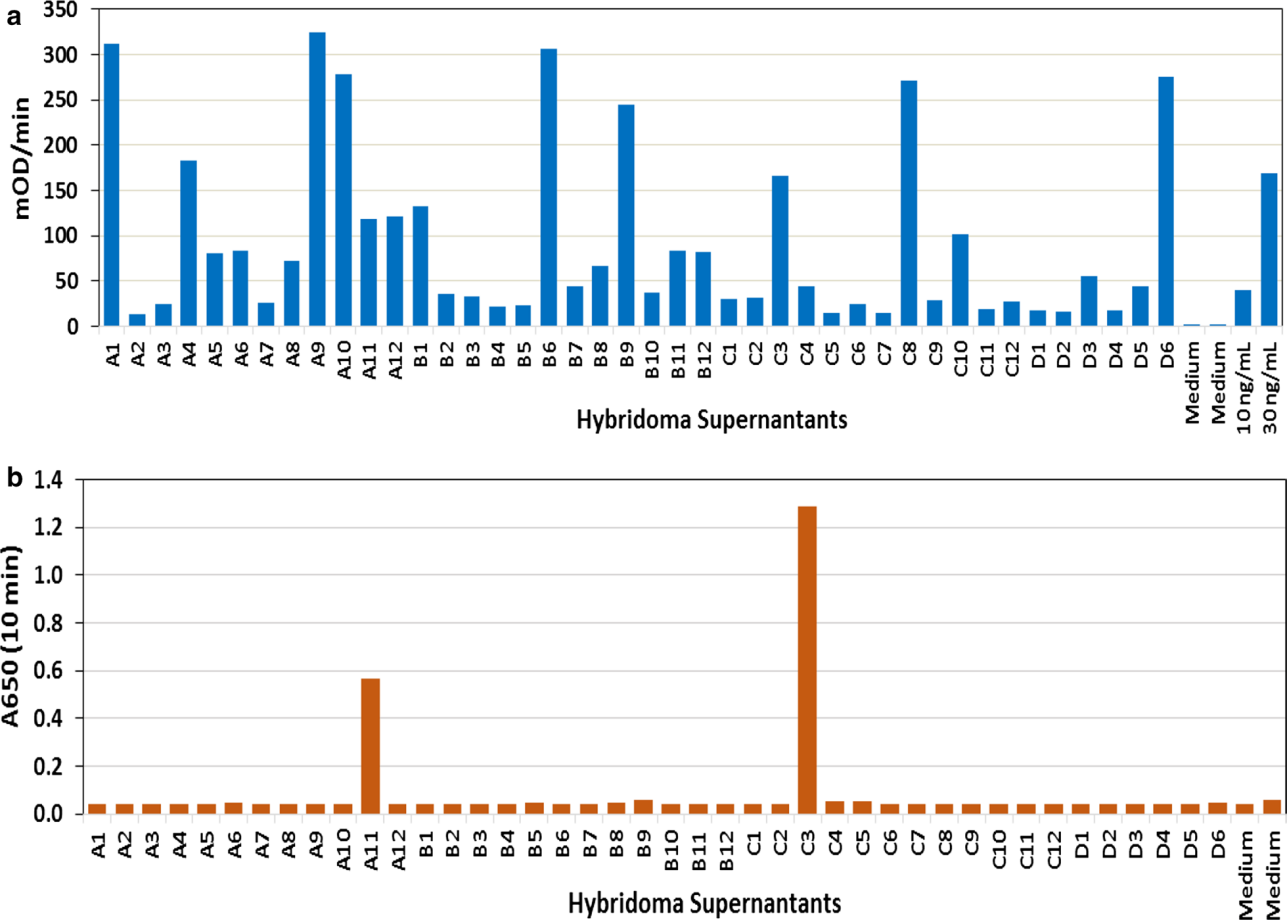
Screening of IgG secreting clones and their binding to vaccine antigens. **a** ELISA screening of a limited set of hybridoma culture supernatant samples identified high numbers of IgG secreting clones. **b** Further analysis of these supernatant samples identified two clones, A11 and C3, for their binding to the mixture of six vaccine peptides

The hybridomas generated from the fusion of CD40L-stimulated cells with plasmacytomas were cultured and their supernatants screened for IgG secretion and vaccine peptide binding by ELISA (Fig. 3). A set of representative data presented in Fig. 3a shows successful creation of a large number of IgG secreting hybridoma clones derived from CD40L-stimulated vaccine-draining lymph node B cells. The culture supernatants from IgG secreting wells were further screened for their binding to the pool of six melanoma peptides identified clones that bind to the vaccine peptides. Figure 3b clearly identified 2 hybridoma clones in this set of representative IgG secreting clones whose culture supernatants bind to the vaccine peptides.

### Specificity of peptide-binding clones

**Fig. 4:**
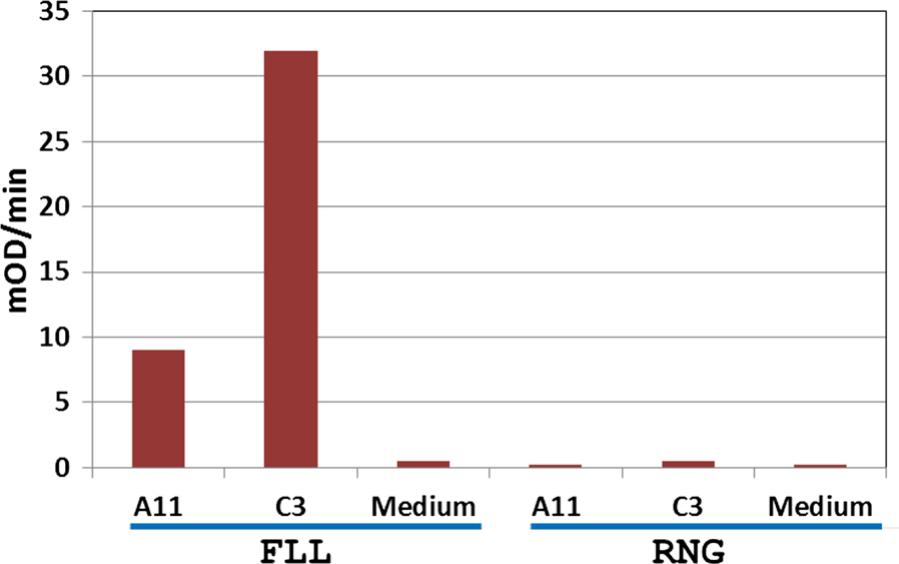
Specificity of the clones that bind to vaccine antigens. Hybridoma culture supernatant samples from clones A11 and C3 specifically bind to FLL peptide and not to RNG peptide

The clones which were found to be positive on screening against the mixture of six peptides were further analyzed by ELISA to identify the target peptide antigen. Both the secreted IgG antibodies from clones A11 and C3 in the representative set which showed binding to a mixture of six melanoma peptides were found to bind FLL peptide. Furthermore, the results also demonstrated the specificity of the antibodies towards FLL peptide as the antibodies from these two clones did not bind to other vaccine peptides, one of which is represented by RNG peptide in Fig. 4.

## Discussion

Our interest is to determine if the vaccine-draining lymph node will be a good source for generating anticancer immune reagents and in particular anti-cancer antibodies. An enabling technology is the ability to remove lymph nodes with exquisite accuracy and in a minimally invasive manner. In human cancer patients, resection of only the first tumor-draining nodes has become standard of care for breast cancer and an expanding list of tumor types [15–17]. The technology involves injection of radioactive colloid around the target tissue which is then carried through the lymphatic vessels to lymph nodes [18]. The first encountered lymph node traps the radioactive colloid. The location of the lymph node is then determined using a hand held gamma detector. The surgeon uses the gamma probe to guide resection of the lymph node. This procedure is now widely available in hospitals and could readily be incorporated into clinical vaccine trials. This technology was used to recover the vaccine draining nodes used for the research reported here.

Multiple studies support the rationale to use lymph nodes that receive drainage from a tumor or a vaccine to better understand the immune response and as a possible source of anti-cancer reagents. For example, T cells recovered from a tumor-draining lymph node in a mouse have been shown to inhibit tumors [19]. The tumor draining T cells were expanded ex vivo and inhibited tumor metastasis when transferred to another mouse. B cells from the tumor-draining lymph node were also shown to inhibit tumors and transfer of both B cells and T cells further enhanced anticancer activity. Multiple studies have demonstrated both in humans and animals that B cells recovered from a tumor-draining lymph node are a source for antitumor antibodies [20–22]. Transfer of serum antibodies induced by a vaccine could inhibit established tumors in a mouse model [8]. B cells recovered from vaccine-draining lymph nodes may be an ideal source for therapeutic antibodies.

Here we determined that B cells recovered from vaccine-draining lymph nodes of human cancer patients secreted antigen-specific antibodies. The results of B cell ELISPOT analyses clearly demonstrated broad B cell responses by the lymph node samples from patients against the vaccine peptides. This appears to be the first time in patients receiving an experimental anti-cancer vaccine that individual B cells derived from the draining lymph node have been shown to secrete IgG antibodies to the vaccine antigen. This observation indicates that the draining lymph node is a potential source of B cells that produce anti-vaccine antibodies. Validation of this observation was achieved by generating hybridomas from the MNCs recovered from the lymph nodes. Multiple fusion events yielded supernatants with antibodies against the vaccine antigens.

In human vaccine trials, beneficial clinical response has been associated with serum titers of vaccine-induced antibodies [6, 7, 23]. Indeed, the patients enrolled in the UVA vaccine study had improved survival associated with elevated anti-vaccine antibodies [2]. If the elevated vaccine-induced antibodies associated with the positive clinical response contributed to improved outcomes, a method to generate these antibodies could be very valuable. We have made a step in the direction toward obtaining such antibodies by demonstrating here that individual B cells that secrete anti-vaccine antibodies were present in vaccine-draining lymph nodes of human cancer patients.

The UVA protocol was designed to preferentially stimulate a T cell response. Despite this T cell vaccine strategy, multiple patients developed serum antibody responses. In all 6 specimens that were analyzed, we were able to recover B cells that secreted anti-vaccine antibodies. Multiple strategies are available that will either enhance or preferentially stimulate a B cell response to a vaccine. Further improvements in the yield of the number of B cells that produce anti-vaccine antibodies are likely by using different vaccine strategies than the one for this study.

We used conventional methods to produce hybridomas by fusing B cells with immortal cells in the last step of our protocol to demonstrate the production of anti-vaccine peptide antibodies. In the hybridoma technology, the number of successful proliferating antibody-secreting cells produced after electrofusion ranges from 1 per 10,000 input MNCs to 1 per 100,000 input MNCs. These methods are extremely inefficient and can be easily replaced by a more efficient method. There are two implications of using hybridoma method for the research presented here. The first is that the number of B cells present in these lymph nodes was sufficiently high that even with the low yield of the hybridoma technique, multiple clones were obtained that produced anti-vaccine antibodies. This is a favorable finding and indicates that a vaccine-draining lymph node may be expected to have ample B cells present that have responded to a vaccine antigen. The other implication is that methods more efficient than conventional hybridoma techniques will likely yield high numbers of vaccine-relevant antibodies. For example, single cell expression cloning the antibody from B cells into proliferating cell lines has a success rate of around 50% [24]. While this strategy requires more stringent upfront selection of candidate B cells than bulk fusion of MNCs, the resulting product of human derived anti-vaccine antibodies justifies such an effort. The potential value of single cell cloning of the antibody gene is quite high. Even a very limited number of B cells that produce anti-vaccine antibodies could provide the starting material for scalable antibody production. We have demonstrated by B cell ELISPOT and confirmed by hybridoma method that B cells are present in human cancer patients that have responded to a cancer vaccine. This confirms the finding of increased serum IgG reactive to these peptides [2]. This information supports further efforts to explore the vaccine-draining lymph node as a source of potentially therapeutic antibodies.

The clinical material used for this study was recovered after 8–14 years of cryopreservation. Despite extended cryopreservation, stimulation with CD40L resulted in 10-fold amplification of CD27^+^ cells. Two rounds of stimulation also increased the proportions of CD19^+^ CD27^+^ cells to 65% from a start of 12% in the sample. Under stimulation, the percentage of class-switched cells, represented by CD19^+^ CD27^+^ IgM^−^ cell subpopulation, approximately doubled. These observations demonstrate that in human cancer patients, cryopreserved B cells remain viable for extended periods of time and when recovered from cryopreservation react appropriately to B cell stimulation protocols. These observations support the utility of vaccine-draining lymph nodes in cancer vaccine trials. It is reasonable to consider adding vaccine-draining lymph node tissue acquisition and cryopreservation to new clinical vaccine trials. This approach will allow controlled use of a rich clinical resource for future research and clinical applications.

## Conclusions

This study successfully demonstrated that in human patients with melanoma, individual B cells recovered from vaccine-draining lymph nodes secreted antibodies to peptide antigens.

This is important since methods that can recover and sequence individual B cells for production of the antibody are becoming more available. The relevance of this approach is that antibodies with the same antigen specificity as serum antibodies could be generated from B cells recovered from a vaccine draining node. It is important that even after long term cryostorage of the clinical material, viable and normally functioning B cells were successfully recovered. The relevance is that archiving this clinical material could be readily accomplished as an adjunct to many different oncologic and infectious disease vaccine trials. Successful recovery of B cells secreting vaccine-specific antibodies in this study, even though the vaccine was designed to favor a T cell response, further supports the broad applicability of this approach.

## Abbreviations

MNCs: mononuclear cells
ELISPOT: enzyme-linked immunospot
